# Mapping the Spatiotemporal Evolution of Emotional Processing: An MEG Study Across Arousal and Valence Dimensions

**DOI:** 10.3389/fnhum.2018.00322

**Published:** 2018-08-10

**Authors:** Charis Styliadis, Andreas A. Ioannides, Panagiotis D. Bamidis, Christos Papadelis

**Affiliations:** ^1^Neuroscience of Cognition and Affection Group, Lab of Medical Physics, School of Medicine, Faculty of Health Sciences, Aristotle University of Thessaloniki, Thessaloniki, Greece; ^2^Laboratory for Human Brain Dynamics, AAI Scientific Cultural Services Ltd., Nicosia, Cyprus; ^3^Laboratory of Children’s Brain Dynamics, Fetal-Neonatal Neuroimaging and Developmental Science Center, Division of Newborn Medicine, Boston Children’s Hospital, Harvard Medical School, Boston, MA, United States

**Keywords:** valence, arousal, temporal windows, international affective picture system, magnetoencephalography

## Abstract

Electrophysiological and functional neuroimaging findings indicate that the neural mechanisms underlying the processing of emotional dimensions (i.e., valence, arousal) constitute a spatially and temporally distributed emotional network, modulated by the arousal and/or valence of the emotional stimuli. We examined the time course and source distribution of gamma time-locked magnetoencephalographic activity in response to a series of emotional stimuli viewed by healthy adults. We used a beamformer and a sliding window analysis to generate a succession of spatial maps of event-related brain responses across distinct levels of valence (pleasant/unpleasant) and arousal (high/low) in 30–100 Hz. Our results show parallel emotion-related responses along specific temporal windows involving mainly dissociable neural pathways for valence and arousal during emotional picture processing. Pleasant valence was localized in the left inferior frontal gyrus, while unpleasant valence in the right occipital gyrus, the precuneus, and the left caudate nucleus. High arousal was processed by the left orbitofrontal cortex, amygdala, and inferior frontal gyrus, as well as the right middle temporal gyrus, inferior parietal lobule, and occipital gyrus. Pleasant by high arousal interaction was localized in the left inferior and superior frontal gyrus, as well as the right caudate nucleus, putamen, and gyrus rectus. Unpleasant by high arousal interaction was processed by the right superior parietal gyrus. Valence was prioritized (onset at ∼60 ms) to all other effects, while pleasant valence was short lived in comparison to unpleasant valence (offsets at ∼110 and ∼320 ms, respectively). Both arousal and valence × arousal interactions emerged relatively early (onset at ∼150 ms, and ∼170 ms, respectively). Our findings support the notion that brain regions differentiate between valence and arousal, and demonstrate, for the first time, that these brain regions may also respond to distinct combinations of these two dimensions within specific time windows.

## Introduction

Neuroimaging and electrophysiological studies contributed to debates over the nature and function of emotion refining our understanding of how the human brain generates and represents emotions. Leaving aside different theories regarding the most appropriate and meaningful conceptualization of emotions (Barrett, 2006; Mauss and Robinson, 2009), neuroscientists converge on the notion that emotional processing involves multiple interrelating brain regions (Lindquist et al., 2012). Though these spatial patterns can overlap for the processing of different emotional stimuli, their associated temporal signature is considered unique (Esslen et al., 2004; Costa et al., 2014; Waugh et al., 2015).

Previously, we examined with magnetoencephalography (MEG) the responses of two brain structures as a function of valence (pleasant/unpleasant) and arousal (high/low) (Russell, 1980): amygdala (Styliadis et al., 2014) which is well-known to be involved in emotional processing, and cerebellum (Styliadis et al., 2015) which is a recent addition to our classical view of the emotion-related distributed circuitry (Adamaszek et al., 2017). In the latter study, we included the time element on a millisecond resolution and extended our current understanding of how valence and arousal are represented in the cerebellar lobules and how their different temporal profiles may be functionally coherent with their emotional role (Styliadis et al., 2015).

The time course of emotions is important due to the different aspects of emotional responses that develop across time (Davidson, 1998). Adding temporal information to the functional maps related to emotional processing can allow tracking the information flow, and the activation of the different regions constituting the emotion-related circuitry. The time-dependent nature of brain regions for different aspects of emotion can provide clues on the way a brain region is selectively engaged in processing specific classes of stimuli. Previous electroencephalographic (EEG) studies assessed the neural representations of basic emotions as dynamic spatiotemporal processes with distinct onset and varying durations (Esslen et al., 2004; Costa et al., 2014). We are motivated by these studies to fill a similar knowledge gap for the spatiotemporal evolution of emotion in terms of valence and arousal.

Functional literature (fMRI and PET) demonstrates that valence and arousal recruit distinct core networks of cortical and subcortical brain regions regardless of the sensory stimuli (Royet et al., 2000; Lewis et al., 2007). EEG studies report that valence and arousal modulate distinct temporal stages of emotional visual processing, with a rather varied and usually early latency range for valence (usually 100–300 ms) and a consistent and late arousal effect (200–1000 ms) (Olofsson et al., 2008). Valence and arousal can interact and therefore be spatially and temporally related (Lewis et al., 2007; Olofsson et al., 2008). Recent EEG evidence demonstrates that the valence by arousal interaction occurs at early (Recio et al., 2014) but also at both early and late temporal stages (Feng et al., 2014), and thus complements previous behavioral (Robinson et al., 2004), fMRI (Lewis et al., 2007; Nielen et al., 2009), EEG, and MEG (Styliadis et al., 2014) findings which indicate that arousal can modulate valence effects.

Previous functional evidence with fMRI and PET did not assess the key element of timing (Waugh and Schirillo, 2012) that is critical in improving our understating about the neural representation of emotions. These techniques offer high spatial resolution of brain activity (Papanicolaou, 1998) and have proven quite successful in identifying the neural substrates of human emotional processing (Phan et al., 2002; Murphy et al., 2003; Wager et al., 2003; Lindquist et al., 2012), despite their limitations in terms of measuring directly the activity of distinct neural populations.

We hypothesize that the processing of emotional visual stimuli activates a set of interacting brain regions, which are selectively activated for the dimensional content of the stimuli at distinct temporal windows. Using MEG, we recorded evoked fields from healthy adult individuals as they passively viewed emotional pictures rated along arousal and valence. A beamformer technique called Synthetic Aperture Magnetometry (SAM) (Robinson and Vrba, 1999) was used to localize the gamma-band responses to the emotional stimuli across time. Gamma-band activity is considered to be particularly important for emotions (Müller et al., 1999; Keil et al., 2001; Oya et al., 2002; Luo et al., 2007, 2009, 2010). Müller et al. (1999) proposed the distribution of gamma oscillations is linked to neural areas engaged in binding emotional information. Keil et al. (2001) reported the enhancement of early mid-gamma band activity (30–45 Hz) at 80 ms post-stimulus in response to aversive stimuli only, while higher gamma activity (46–65 Hz) at 500 ms showed an enhancement of arousing, compared to neutral visual stimuli. In addition, Luo et al. (2007) used similar techniques to the ones reported in the current study and demonstrated gamma band activity within the amygdala, visual cortex, and inferior frontal gyrus (IFG)/insula in response to emotional stimuli. Our data were partially published in two previous studies of our group (Styliadis et al., 2014, 2015) focusing on the specific role of the sub-divisions of amygdala (Styliadis et al., 2014), and cerebellum (Styliadis et al., 2015) in the emotional processing of arousal and valence. Here, we examine brain responses to emotional stimuli considering the entire cerebrum. We aim to map the spatiotemporal stream of brain activity in response to passive viewing of emotional pictures balanced in terms of valence and arousal. We proceed to: (i) localize the brain regions that sub-serve the processing of valence and arousal, (ii) capture the onset of the processing of the different levels of valence and arousal, and (iii) track their evolution (onset and offset times) as a function of time.

## Materials and Methods

### Participants

Our sample consisted of ten healthy adults (five males, mean age 30.6 ± 6.34, 23–40 years, five females mean age 27.8 ± 5.3, 21–35 years). We confirmed the absence of any health issue or use of prescription medications or drugs with the use of a short questionnaire. No previous history of neurological or psychiatric illness was reported by the participants. Similarly, the participants self-reported to have normal or corrected-to-normal vision. The participants received adequate information on the stimuli type and modality. Then, they gave their written informed consent in compliance with the Code of Ethics of the World Medical Association (Declaration of Helsinki) and the standards established by the host institution’s (Brain Science Institute, RIKEN) ethics committee. The approval for the study was given by the RIKEN Research Ethics Committee, Wako Third Committee 15-25(10). As a measure for ensuring the effectiveness of the erotic stimuli, we recruited only self-identified heterosexual individuals (see section “Balancing of Stimuli”). Exclusion criteria were as follows: (i) history of medical illness (i.e., psychiatric, neurological, and physical), (ii) any reported drug or alcohol abuse, (iii) prescription medications, and (iv) the existence of metal implants such as dental crowns, which affect the magnetic evoked fields. The participants were asked to abstain from alcohol and caffeine the day before and the day of the experiment. We informed the participants that they could terminate their participation at any time without the need to provide any justification for their decision (no participants withdrew consent). As indicated in previous studies using the same dataset (Styliadis et al., 2014, 2015), we originally recruited 12 subjects but two male subjects were removed from the analysis due to heavy artifact contamination.

### Emotional Stimuli

**FIGURE 1:**
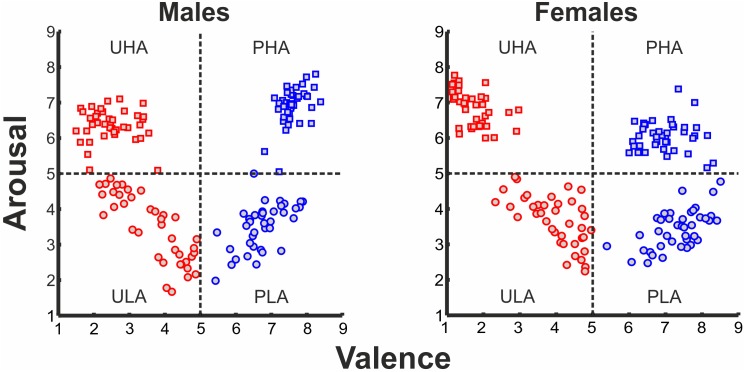
Ratings of IAPS affective pictorial stimuli for arousal and valence (left panel: ratings of males, right panel: ratings of females) forming a 2D affective space. Normative valence and arousal on the *x*- and *y*-axis, respectively. Red rectangles, UHA stimuli; red circles, ULA stimuli; blue rectangles, PHA stimuli; blue circles, PLA stimuli.

One hundred and sixty stimuli were selected from the International Affective Picture System (IAPS) (Lang et al., 2008). These stimuli are distributed in the two-dimensional affective space, based on co-varying normative ratings of valence and arousal. The selected IAPS stimuli were categorized into four categories^1^: (i) pleasant with high arousal (PHA), (ii) pleasant with low arousal (PLA), (iii) unpleasant with high arousal (UHA), and (iv) unpleasant with low arousal (ULA). The four emotion categories feature in total a broad range of stimulus types. For instance, pleasant with high arousal (PHA) features sports, and erotica themes (the percentage of erotic pictures at the subject level for the PHA category is 70% for males and 47.5% for females), pleasant with low arousal (PLA) consists of neutral faces, and scenes of nature, unpleasant with high arousal (UHA), features human violence, mutilation, attack and angry faces, and unpleasant with low arousal (ULA) has scenes of pollution and disease. The distribution of IAPS pictures in the 2D affective space is illustrated in **Figure 1**. Given that valence and arousal interact (Cuthbert et al., 2000; Robinson et al., 2004), we considered the possible interaction between the levels of valence and arousal.

### Balancing of Stimuli

To ensure that possible differences in the evoked responses to the different levels of valence and arousal of the emotional stimuli are generated by the emotional content of the stimuli and not by incidental visual properties or gender differences, we balanced our stimuli for complexity, overall Apparent Contrast (AC) and AC for each color level between males and females, spatial frequencies and the AC of each picture for all the participants (Delplanque et al., 2007), as well as for both genders in terms of arousal and valence ratings. Details regarding our approach to effectively balance the stimuli can be found elsewhere (Styliadis et al., 2015). In short, regarding the control of gender differences, our stimuli categorization was based on the gender-specific ratings provided by the IAPS collection in accordance with previous studies by our group (Lithari et al., 2010; Styliadis et al., 2014). *T*-tests indicated that there were no differences (*p* > 0.05) between the ratings of the two sets of stimuli (one for males and the other for females) for both valence and arousal. **Table 1** lists the gender-specific ratings for the selected stimuli as provided by the IAPS collection as well as our own participants’ group ratings. We explored potential main as well as valence × arousal interaction effects in the ratings of the stimuli for both normative and participant data and for all subjects as well as both genders separately. Therefore, we performed a multifactorial ANOVA having the ratings for valence and arousal as dependent variables and the levels of valence (unpleasant and pleasant) and arousal (low and high) as independent variables (**Tables 1, 2**). As previously noted, we recruited only self-identified heterosexual individuals to ensure the effectiveness of the erotic stimuli. The IAPS collection contains erotic pictures of naked males and females as well as heterosexual couples. The content of the selected erotic pictures fits with the sexual targets of heterosexual men and women. Heterosexual and homosexual males have been previously reported to exhibit clear category specific arousal with a high degree of correspondence to their self-reported sexual preference. That is, both heterosexual and homosexual men tend to show substantial and little arousal to erotic stimuli depicting their preferred and non-preferred sex, respectively. Though heterosexual women do not show this category specificity, in favor of male versus female stimuli, this lack of specificity is due to their equal appraisals of both male and female stimuli (Chivers et al., 2004). Considering the gender-specific normative ratings provided in the IAPS database, we chose erotic stimuli that were perceived as pleasant (a bit to most) and arousing (a bit to very) by males and females rating their preferred sex and thus limited the stimuli use to a heterosexual group. A neuroimaging study (Ponseti et al., 2006) demonstrated that men and women, regardless of sexual preference, exhibit identical patterns of neural activation in response to visual sexual stimuli. Though both heterosexual and homosexual men and women activated brain areas related to reward, the activation of the reward system was highest when viewing pictures of their preferred sex. This study supports our approach as males and females differ in the stimuli and strategies that activate the neural pathways underlying sexual arousal. Regarding the stimuli control for avoiding any possible effects of the physical properties of the stimuli confounding our findings, we followed the procedure described by Delplanque et al. (2007)^2^. The considered effects of the stimuli’ visual properties were non-significant with respect to the experimental conditions for both gender groups (*p* > 0.05).

**Table 1:** Mean (SD) normative and participants’ ratings for valence, arousal, and their interactions.

		Normative ratings	Participants’ ratings
**All subjects**			
Valence	Pleasant	7.16 (±0.67)	7.01 (±0.44)
	Unpleasant	2.94 (±1.29)	3.25 (±1.33)
Arousal	High arousing	6.54 (±0.62)	6.66 (±0.60)
	Low arousing	3.53 (±0.80)	4.16 (±0.57)
Valence by arousal interaction	Valence for PHA	7.30 (±0.59)	7.03 (±0.48)
	Valence for PLA	7.03 (±0.70)	6.98 (±0.45)
	Valence for UHA	2.09 (±0.67)	2.69 (±1.20)
	Valence for ULA	3.80(±0.83)	3.82 (±0.98)
	Arousal for PHA	6.48 (±0.64)	6.60 (±0.90)
	Arousal for UHA	6.61 (±0.54)	6.73 (±1.03)
	Arousal for PLA	3.48 (±0.58)	4.12 (±0.70)
	Arousal for ULA	3.59 (±0.84)	4.20 (±0.72)
**Males**			
Valence	Pleasant	7.18 (±0.66)	6.97 (±0.61)
	Unpleasant	3.05 (±0.96)	3.45 (±1.30)
Arousal	High arousing	6.64 (±0.56)	6.52 (±0.83)
	Low arousing	3.51 (±0.80)	4.21 (±0.91)
Valence by arousal interaction	Valence for PHA	7.62 (±0.34)	7.06 (±0.49)
	Valence for PLA	6.74 (±0.59)	6.88 (±0.47)
	Valence for UHA	2.49 (±0.62)	3.10 (±1.14)
	Valence for ULA	3.62 (±0.62)	3.80 (±0.97)
	Arousal for PHA	6.92 (±0.51)	6.63 (±0.57)
	Arousal for UHA	6.36 (±0.42)	6.41 (±0.62)
	Arousal for PLA	3.53 (±0.62)	4.26 (±0.84)
	Arousal for ULA	3.48 (±0.42)	4.16 (±0.82)
**Females**			
Valence	Pleasant	7.15 (±0.67)	7.05 (±0.59)
	Unpleasant	2.83 (±1.29)	3.07 (±1.51)
Arousal	High arousing	6.46 (±0.62)	6.81 (±1.36)
	Low arousing	3.56 (±0.65)	4.12 (±0.67)
Valence by arousal interaction	Valence for PHA	6.98 (±0.62)	7.02 (±0.47)
	Valence for PLA	7.33 (±0.68)	7.08 (±0.41)
	Valence for UHA	1.71 (±0.47)	2.28 (±1.13)
	Valence for ULA	3.98 (±0.71)	3.86 (±1.02)
	Arousal for PHA	6.05 (±0.42)	6.57 (±1.14)
	Arousal for UHA	6.87 (±0.50)	7.05 (±1.25)
	Arousal for PLA	3.43 (±0.54)	3.99 (±0.51)
	Arousal for ULA	3.70 (±0.70)	4.25 (±0.62)

*T-tests were performed to ensure that there were no significant differences between the pictures used for males and those for females.*

**Table 2:** Stimuli characteristics for valence and arousal main effects and interactions at *p* < 0.05.

Dependent variable	Independent variable		
	Valence	Arousal	Valence × Arousal
**Normative ratings for all subjects**			
Valence ratings	*F*(1, 316) = 2852.378, Wilks’ Λ (2,315) = 0.099	n.s.	*F*(1, 316) = 155.534, Wilks’ Λ (2,315) = 0.666
Arousal ratings	*F*(1, 316) = 82.598, Wilks’ Λ (2,315) = 0.160	*F*(1, 316) = 1650.859, Wilks’ Λ (2,315) = 0.160	n.s.
**Participants’ ratings for all subjects**			
Valence ratings	*F*(1, 316) = 1545.850 Wilks’ Λ (2,315) = 0.167	n.s.	*F*(1, 316) = 39.435 Wilks’ Λ (2,315) = 0.887
Arousal ratings	*F*(1, 316) = 32.076 Wilks’ Λ (2,315) = 0.316	*F*(1, 316) = 682.584 Wilks’ Λ (2,315) = 0.316	n.s.
**Normative ratings for males**			
Valence ratings	*F*(1, 156) = 1623.907, Wilks’ Λ (2,155) = 0.08	*F*(1, 156) = 8.145, Wilks’ Λ (2,155) = 0.08	*F*(1, 156) = 96.715, Wilks’ Λ (2,155) = 0.562
Arousal ratings	n.s.	*F*(1, 156) = 881.907, Wilks’ Λ (2,155) = 0.145	*F*(1, 156) = 6.008, Wilks’ Λ (2,155) = 0.562
**Participants’ ratings for males**			
Valence ratings	*F*(1, 156) = 715.358, Wilks’ Λ (2,155) = 0.172	n.s.	*F*(1, 156) = 11.337, Wilks’ Λ (2,155) = 0.925
Arousal ratings	*F*(1, 156) = 3.990, Wilks’ Λ (2,155) = 0.278	*F*(1, 156) = 402.714, Wilks’ Λ (2,155) = 0.278	n.s.
**Normative ratings for females**			
Valence ratings	*F*(1, 156) = 1855.164, Wilks’ Λ (2,155) = 0.078)	*F*(1, 156) = 37.866, Wilks’ Λ (2,155) = 0.078	*F*(1, 156) = 92.620, Wilks’ Λ (2,155) = 0.620
Arousal ratings	*F*(1, 156) = 170.912, Wilks’ Λ (2,155) = 0.11	*F*(1, 156) = 1082.221, Wilks’ Λ (2,155) = 0.119	*F*(1, 156) = 9.958 Wilks’ Λ (2,155) = 0.620
**Participants’ ratings for females**			
Valence ratings	*F*(1, 156) = 917.916, Wilks’ Λ (2,155) = 0.145	*F*(1, 156) = 6.142, Wilks’ Λ (2,155) = 0.145	*F*(1, 156) = 32.990, Wilks’ Λ (2,155) = 0.825
Arousal ratings	*F*(1, 156) = 38.678, Wilks’ Λ (2,155) = 0.311	*F*(1, 156) = 324.639, Wilks’ Λ (2,155) = 0.311	n.s.

### Experimental Procedure

The experimental procedure was performed in a dimly lit Magnetically Shielded Room (MSR). The stimuli were back-projected onto a 10-inch MEG compatible screen via a DLP projector with a 96 Hz refresh rate (HL8000Dsx+, NEC Viewtechnology Ltd., Japan) located outside the MSR. These projections were delivered at 55 cm from the participant’s eyes, at a visual angle of 4° horizontally and vertically and were controlled by the Presentation software (Neurobehavioral Systems, Inc., United States). Markers in the MEG data were synchronized to the onset of each visual stimulus via a photodiode signal.

The stimuli were presented in a random sequence, within two runs (160 trials, 40 trials per category), to avoid possible habituation to emotional stimulation. Stimuli and inter-stimuli (fixation cross) were projected centered on a homogenous black background. Each run began with the projection of a fixation cross, at 40 × 40 pixels resolution for a pseudo-randomized interval of 1500 ± 200 ms. This interval between stimuli allowed for the participant’s emotional disengagement from the previous emotional stimulus and his/her physiological shift to the pre-stimulus level. Trials were projected at 400 × 400 pixels resolution for 1000 ms along with the fixation cross. The trial duration was regarded short enough so as not to produce considerable variability in the participants’ responses. The trial duration was regarded long enough to engage processes that have a relatively fast time constant and elicit responses in the affective neural substrates. Each run lasted 220 s resulting in a total recording time of 440 s.

After the MEG recordings, each participant rated the stimuli with the Self-Assessment Manikins model (Bradley and Lang, 1994) on a scale of 1–9 for valence (1 = most unpleasant, 2 = fairly unpleasant, 3 = somewhat unpleasant, 4 = a bit unpleasant, 5 = neutral, 6 = a bit pleasant, 7 = somewhat pleasant, 8 = fairly pleasant, 9 = most pleasant) and arousal (1 = very passive/calm, 2 = fairly passive/calm, 3 = somewhat passive/calm, 4 = a bit calm 5 = not calm nor at all excited, 6 = a bit aroused, 7 = somewhat active/aroused, 8 = fairly active/aroused, 9 = very excited/aroused). The participants’ ratings and the normative IAPS ratings were compared with the use of a *t*-test. No significant differences (*p* > 0.05) were found between these ratings (**Table 1**).

### Co-registration

We obtained anatomical MRIs (1.5 T MRI, Model ExcelArt, Toshiba Medical Systems) for each participant, using a T1-weighted volume acquisition sequence resulting in a voxel-size of 1 mm × 1 mm × 1 mm. The head position of each participant was registered twice (start and end of each measurement) with the use of five localization coils (three in nasion, left and right pre-auricular points and two on the forehead). Head movements did not exceed 5 mm. The co-registration was accurate when the mean distance between the surface of the head and the face, derived from the 3D camera system, the 3D digitizer, and the anatomical image, was less than 2 mm. The co-registration between the MEG and MRI was described in Papadelis et al. (2011).

### Data Acquisition

The MEG recordings were performed at a sampling rate of 1250 Hz using a 151-channel CTF whole head MEG system (VSM MedTech Ltd.) with a band-pass of DC to 200 Hz. The CTF MEG system features synthetic third gradient balancing, namely an active noise cancelation technique that uses reference channels to subtract background interference. The participants were positioned comfortably in a seated orientation with their head located in the dewar to reduce postural muscle artifacts. The participants were requested to avoid movement, eye movements, and eye-blinks during the trials and to focus on the fixation cross. Co-current electrooculographic (EOG) and electrocardiographic (ECG) recordings were performed with the use of four and five Ag/AgCl electrodes, respectively.

### Data Pre-processing

We removed the DC offset. We inspected the raw data off-line for bad channels. We applied automated threshold procedures to the MEG signals to reject off-line any trials contaminated with muscle artifacts, signal jumps or distortions of the magnetic field. Two bad channels were identified and subsequently the corresponding trials were removed from the final analysis. The remaining trials with field magnitudes less than 1 × 10^-11^ Tesla in any channel were kept for further analysis. We manually inspected the MEG and EOG signals to make sure that the artifact rejection performance was good. We applied a second order Butterworth notch filter to remove the line noise (50 Hz and its harmonics). We removed artifactual signal components in MEG data (blinks, facial muscle components, and cardiac artifacts) with the use of the independent component analysis (ICA) using the Brain Electrical Source Analysis software (BESA Research, version 6.0, Megis Software). We reduced the dimensionality of the data by principal component analysis (PCA) before the calculation of ICA. All PCA components that explain less than 1% variance were ignored.

### MEG Source Analysis

We created a multi-sphere head model per participant using his/her anatomical MRI. SAM (Robinson and Vrba, 1999) was used to spatially map task-related power changes in oscillatory brain activity across participants in the gamma frequency band (30–100 Hz). SAM employs an optimal spatial filter for each voxel in the brain which links activity in each voxel to the MEG sensor array. This spatial filter estimates a measure of source power in each voxel as a function of time and is constructed using the weighted sum of all MEG sensors. We employed the dual-state SAM imaging approach. This approach calculates the change of task-related power between the active and control states (time windows) divided by MEG sensor noise projected through the beamformer to obtain pseudo-t values. SAM requires no *a priori* assumptions as to the number of sources activated and is ideally suited for the analysis of induced activity. The SAM output for PHA, PLA, UHA, and ULA conditions per participant was the difference between the active and the control state. A single SAM image per participant, per condition, was generated by averaging the corresponding SAM images from the two runs. The resulting SAM images were overlaid on the individual MRI. In order to assess the spatiotemporal evolution of emotional visual processing, that is at what time significant gamma band oscillations emerge, peak, and offset, and in which brain regions, we used the sliding window approach for SAM analysis (Luo et al., 2010; Styliadis et al., 2015). This analysis uses an active window of 1000 ms sliding with a step of 10 ms (i.e., -1000 to 0 ms, -990 to 10 ms, -980 to 20 ms,…, -10 to 990 ms, 0 to 1000 ms), and a passive (control) window of 1000 ms prior to stimulus onset (-1000 to 0 ms). The concatenation of the resulting SAM images provided the temporal sequence of spatial maps across all time points from 0 to 1000 ms.

### Group Analysis of MEG Source Activity

We normalized the anatomical image of the participants and their corresponding SAM images for each condition into the Montreal Neurological Institute (MNI) space with the segmentation module of SPM8^3^. We performed the statistical group analysis of the normalized SAM images also with SPM8. We designed a factorial model to explore the main effects of valence and arousal and the valence × arousal interaction effect using the second level of SPM analysis. This model is equivalent to a 2 × 2 model of repeated ANOVA measures with arousal (high/low) and valence (pleasant/unpleasant) as the within-subject factors. The mean activation across participants for valence, arousal, and the valence × arousal interaction was constrained in the cerebrum. If a significant main effect or interaction was revealed, *post hoc* comparisons using *t*-test were performed between the different levels of the significant factor. We applied a permutation method for peak-cluster level error correction (AlphaSim) at 1% level to the statistical results, as implemented in REST software (Song et al., 2011), by taking into account the significance threshold, *p* < 0.001 uncorrected, and cluster size (threshold size, 157 voxels), thereby controlling for multiple comparisons in the spatial domain. We did not adjust the multiple ANOVA’s *p*-values to control for the multiple comparison problem along the temporal domain.

### Probabilistic Maps

Regions of significant activation were identified using probabilistic cytoarchitectonic maps (PCMs) (Amunts et al., 2007; Zilles and Amunts, 2010). Their use allowed us to assign the activation sites to histologically defined brain regions. The PCMs are freely available through the anatomy toolbox (Eickhoff et al., 2005)^4^. Thus, we assigned a cytoarchitectonic identity to our activation sites based on the anatomical probabilities provided in the toolbox. The algorithm used (Eickhoff et al., 2006), assigns each voxel to the most probable cytoarchitectonic area at the position under investigation.

## Results

### Stimuli Characteristics

#### Normative Ratings

##### All subjects

The comparison between cells of the factor valence showed that the normative valence ratings for all subjects were significantly higher for positively valenced stimuli [*F*(1, 316) = 2852.378, *p* < 0.05; Wilks’ Λ (2,315) = 0.099] but did not differ significantly between cells of the factor arousal. The same analysis showed that the normative arousal ratings for all subjects were significantly higher for negatively valenced stimuli [*F*(1, 316) = 82.598, *p* < 0.05; Wilks’ Λ (2,315) = 0.160], and significantly higher for high arousing stimuli [*F*(1, 316) = 1650.859, *p* < 0.05; Wilks’ Λ (2,315) = 0.160]. Also, there was a significant valence × arousal interaction for the normative valence ratings [*F*(1, 316) = 155.534, *p* < 0.05; Wilks’ Λ (2,315) = 0.666], but not for the arousal ratings (**Tables 1, 2**).

##### Male subjects

The comparison between cells of the factor valence showed that the male normative valence ratings were significantly higher for positively valenced stimuli [*F*(1, 156) = 1623.907, *p* < 0.05; Wilks’ Λ (2,155) = 0.08] and also significantly higher for high arousing stimuli [*F*(1, 156) = 8.145, *p* < 0.05; Wilks’ Λ (2,155) = 0.08]. The male normative arousal ratings did not differ significantly between cells of the factor valence, but were significantly higher for high arousing stimuli [*F*(1, 156) = 881.907, *p* < 0.05; Wilks’ Λ (2,155) = 0.145]. Also, there was a significant valence × arousal interaction for normative valence ratings [*F*(1, 156) = 96.715, *p* < 0.05; Wilks’ Λ (2,155) = 0.562], and for the arousal ratings [*F*(1, 156) = 6.008, *p* < 0.05; Wilks’ Λ (2,155) = 0.562] (**Tables 1, 2**).

##### Female subjects

Female normative valence ratings were significantly higher for positively valenced stimuli [*F*(1, 156) = 1855.164, *p* < 0.05; Wilks’ Λ (2,155) = 0.078], and significantly higher for low arousing stimuli [*F*(1, 156) = 37.866, *p* < 0.05; Wilks’ Λ (2,155) = 0.078]. Female normative arousal ratings were significantly higher for negatively valenced stimuli [*F*(1, 156) = 170.912, *p* < 0.05; Wilks’ Λ (2,155) = 0.119], and also significantly higher for high arousing stimuli [*F*(1, 156) = 1082.221, *p* < 0.05; Wilks’ Λ (2,155) = 0.119]. Finally, there was a significant valence × arousal interaction for the normative valence ratings [*F*(1, 156) = 92.620, *p* < 0.05; Wilks’ Λ (2,155) = 0.620], and also for the arousal ratings [*F*(1, 156) = 9.958, *p* < 0.05; Wilks’ Λ (2,155) = 0.620] (**Tables 1, 2**).

#### Participants’ Ratings

##### All subjects

The comparison between cells of the factor valence showed that the participants’ valence ratings for all subjects were significantly higher for positively valenced stimuli [*F*(1, 316) = 1545.850, *p* < 0.05; Wilks’ Λ (2,315) = 0.167] but did not differ significantly between cells of the factor arousal. The participants’ arousal ratings for all subjects were significantly higher for negatively valenced stimuli [*F*(1, 316) = 32.076, *p* < 0.05; Wilks’ Λ (2,315) = 0.316], and significantly higher for high arousing stimuli [*F*(1, 316) = 682.584, *p* < 0.05; Wilks’ Λ (2,315) = 0.316]. Also, there was a significant valence × arousal interaction for the participants’ valence ratings [*F*(1, 316) = 39.435, *p* < 0.05; Wilks’ Λ (2,315) = 0.887], but not for the arousal ratings (**Tables 1, 2**).

##### Male subjects

The comparison between cells of the factor valence showed that the male participants’ valence ratings were significantly higher for positively valenced stimuli [*F*(1, 156) = 715.358, *p* < 0.05; Wilks’ Λ (2,155) = 0.172] but did not differ significantly between cells of the factor arousal. Male participants’ arousal ratings were significantly higher (marginally) for negatively valenced stimuli [*F*(1, 156) = 3.990, *p* < 0.05 (=0.048); Wilks’ Λ (2,155) = 0.278], and also significantly higher for high arousing stimuli [*F*(1, 156) = 402.714, *p* < 0.05; Wilks’ Λ (2,155) = 0.278]. Also, there was a significant valence × arousal interaction for the participants’ valence ratings [*F*(1, 156) = 11.337, *p* > 0.05; Wilks’ Λ (2,155) = 0.925], but not for the arousal ratings (**Tables 1, 2**).

##### Female subjects

Female participants’ valence ratings were significantly higher for positively valenced stimuli [*F*(1, 156) = 917.916, *p* < 0.05; Wilks’ Λ (2,155) = 0.145] and also significantly higher for low arousing stimuli [*F*(1, 156) = 6.142, *p* < 0.05; Wilks’ Λ (2,155) = 0.145]. Female participants’ arousal ratings were significantly higher for negatively valenced stimuli [*F*(1, 156) = 38.678, *p* < 0.05; Wilks’ Λ (2,155) = 0.311], and also significantly higher for high arousing stimuli [*F*(1, 156) = 324.639, *p* < 0.05; Wilks’ Λ (2,155) = 0.311]. Finally, there was a significant valence × arousal interaction for the participants’ valence ratings [*F*(1, 156) = 32.990, *p* < 0.05; Wilks’ Λ (2,155) = 0.825], but not for the arousal ratings (**Tables 1, 2**).

### Source Reconstruction

**FIGURE 2:**
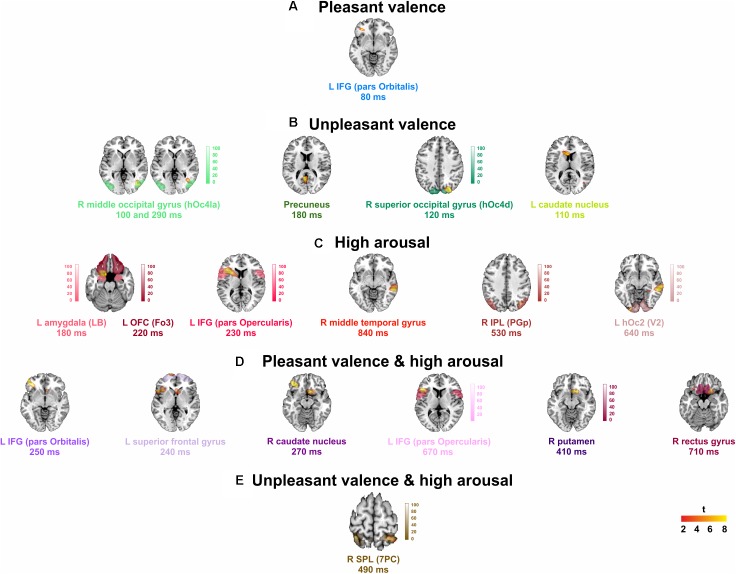
Group analysis activations at their peak for: **(A)** pleasant, and **(B)** unpleasant valence, **(C)** high arousal stimuli, and **(D)** pleasant, and **(E)** unpleasant valence interaction to high arousal. L, left; R, right. Points counted as left or right were at least 6 mm from the midline. Red to yellow color scale, *t*-values. Threshold: AlphaSim corrected at *p* < 0.05 by taking into account the significance threshold, *p* < 0.001 uncorrected, and cluster size (threshold size, 157 voxels). Significant activations are overlaid by the corresponding cytoarchitectonic regions (when available).

Significant spatial maps are reported at *p* < 0.05 AlphaSim corrected significance level (**Figure 2** and **Table 3**). Pleasant valence was localized in the left IFG (pars Orbitalis). The effect of unpleasant valence was localized in the right occipital gyrus [middle (hOc4la) and superior (hOc4d)], and in the precuneus and left caudate nucleus. High arousal was processed by left orbitofrontal cortex OFC (Fo3), left amygdala (LB), left IFG (pars Opercularis), right middle temporal gyrus, right inferior parietal lobule (IPL) (PGp), and left hOc2 (V2). We did not find any significant activity for low arousal. The pleasant valence by high arousal interaction was localized in IFG (pars Orbitalis and pars Opercularis), left superior frontal gyrus, right caudate nucleus, right putamen, and right gyrus rectus. The unpleasant valence by high arousal interaction was localized in the right superior parietal gyrus (SPL) (PC).

**Table 3:** Local statistical maxima for sliding window analysis.

	H	MNI coordinates (mm)			Onset (ms)	Maxima (ms)	Offset (ms)	*T* (at maxima)	CS	Cluster-level corrected *p*-value
		*x*	*y*	*z*						
**Pleasant**										
IFG (pars Orbitalis)	L	-36	38	-8	60	80	110	3.94	162	0.040
**Unpleasant**										
Middle occipital gyrus (hOc4la)	R	40	-76	4	60	100	130	5.30	181	0.046
Middle occipital gyrus (hOc4la)	R	42	-70	4	260	290	320	4.19	169	0.048
Precuneus		-2	-62	28	70	180	260	5.03	289	0.016
Superior occipital gyrus (hOc4d)	R	30	-80	44	80	120	250	4.57	218	0.032
Caudate nucleus	L	-10	12	16	90	110	140	3.94	294	0.006
**High arousal**										
OFC (Fo3)	L	-22	8	-18	150	220	550	5.08	314	0.020
Amygdala (LB)	L	-16	-7	-19	160	180	340	4.76	314	0.042
IFG (pars Opercularis)	L	-50	10	14	170	230	260	5.26	412	0.021
Middle temporal gyrus	R	60	-44	-4	410	840	1000	4.93	406	0.021
IPL (PGp)	R	42	-76	42	470	530	840	4.83	194	0.037
hOc2 (V2)	L	-28	-91	-6	540	640	780	4.95	173	0.048
**Pleasant valence and high arousal**										
IFG (pars Orbitalis)	L	-48	40	-2	170	250	280	5.03	188	0.044
Superior frontal gyrus	L	-12	62	3	210	240	400	4.90	166	0.049
Caudate nucleus	R	8	8	-10	220	270	300	4.78	310	0.035
IFG (pars Opercularis)	L	-48	18	12	270	670	1000	4.92	310	0.014
Putamen	R	16	12	-8	330	410	500	4.38	256	0.042
Gyrus rectus	R	22	12	-14	640	710	820	4.62	286	0.040
**Unpleasant valence and high arousal**										
SPL (7PC)	R	30	-58	70	350	490	640	4.65	203	0.034

*Activated cerebral regions for valence (pleasant and unpleasant), high arousal, (pleasant and unpleasant) valence by high arousal interaction at p < 0.05 AlphaSim corrected. Results are superimposed on standardized MNI coordinates; H, hemisphere; L, left; R, right; T, oxt-values for each peak, CS, cluster size in number of activated voxels along the significant time course; Cluster corrected oxp-value is the minimal oxp-value along the significant time course.*

### Timing Evolution

**FIGURE 3:**
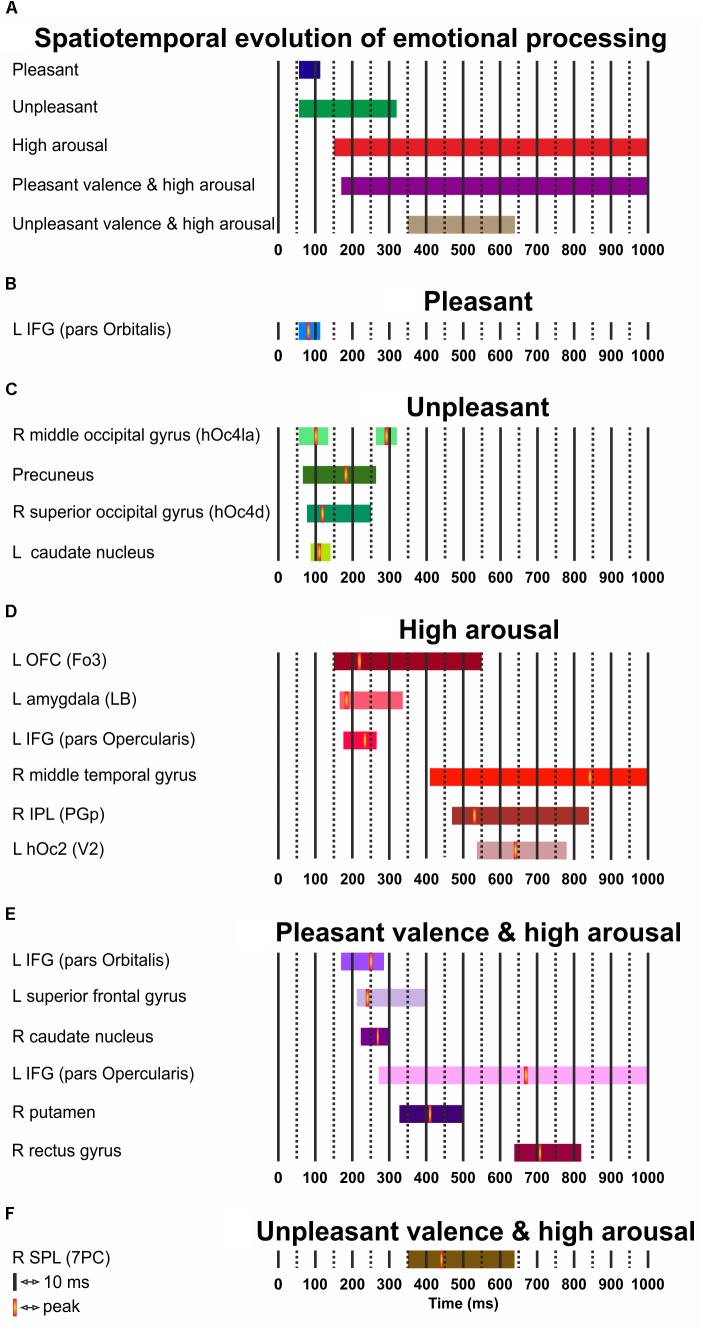
**(A–F)** Spatiotemporal cerebral activity for valence (pleasant and unpleasant), high arousal, and the (pleasant and unpleasant) valence interaction by high arousal for a period of 1000 ms. L, left; R, right.

**Figure 3** presents the temporal cerebral activity for valence (pleasant/unpleasant), high arousal, and the valence (pleasant/unpleasant) interaction to high arousal within 1000 ms. Pleasant activity in the left IFG (pars Orbitalis) had an early onset at ∼60 ms, peaked at ∼80 ms, and had an offset at ∼110 ms. Unpleasant valence was initially attributed to right middle occipital gyrus (hOc4la), which had an early onset at ∼60 ms, peaked at ∼100 ms, and lasted up to ∼130 ms. Unpleasant valence was then processed in the precuneus (onset at ∼70 ms, peak at ∼180 ms, offset at ∼260 ms), the right superior occipital gyrus (hOc4d) (onset at ∼80 ms, peak at ∼120 ms, offset at ∼250 ms) and the left caudate nucleus (onset at ∼90 ms, peak at ∼110 ms, offset at ∼140 ms). At a later stage, unpleasant valence was processed again by the right middle occipital gyrus (hOc4la) (onset at ∼260 ms, peak at ∼290 ms, offset at ∼320 ms). High arousal was initially attributed to the left OFC (Fo3) (onset at ∼150 ms, peak at ∼220 ms, offset at ∼550 ms), and the amygdala (LB) (onset at ∼160 ms, peak at ∼180 ms, offset at ∼340 ms). High arousal was then processed by the IFG (pars Opercularis) (onset at ∼170 ms, peak at ∼230 ms, offset at ∼260 ms), the right middle temporal gyrus (onset at ∼410 ms, peak at ∼840 ms, offset at ∼1000 ms), the IPL (PGp) (onset at ∼470 ms, peak at ∼530 ms, offset at ∼840 ms), and the left hOc2 (V2) (onset at ∼540 ms, peak at ∼640 ms, offset at ∼780 ms). Pleasant valence by high arousal interaction was initially attributed to the left IFG (pars Orbitalis) (onset at ∼170 ms, peak at ∼250 ms, offset at ∼280 ms), the left superior frontal gyrus (onset at ∼210 ms, peak at ∼240 ms, offset at ∼400 ms), and the right caudate nucleus (onset at ∼220 ms, peak at ∼270 ms, offset at ∼300 ms). The interaction was processed again in the left IFG (pars Opercularis) (onset at ∼270 ms, peak at ∼670 ms, offset at ∼1000 ms), right putamen (onset at ∼330 ms, peak at ∼410 ms, offset at ∼500 ms) and right gyrus rectus (onset at ∼640 ms, peak at ∼710 ms, offset at ∼820 ms). Unpleasant valence by high arousal interaction was processed by the right SPL (PC) (onset at ∼350 ms, peak at ∼490 ms, offset at ∼640 ms).

## Discussion

We mapped the spatiotemporal evolution of emotional visual processing as a function of arousal and valence by examining modulations of MEG activity in the gamma band during the passive viewing of emotional pictures. We found that: (i) valence and arousal are represented by mainly dissociable neural substrates; (ii) some brain regions [IFG (pars Orbitalis/pars Opercularis)] respond independently to both valence and arousal; and (iii) these neuronal representations unfold in parallel along distinct temporal streams, which depend on valence and arousal.

### Dissociable Valence and Arousal Neural Substrates

#### Valence Encoding

Our results agree with previous PET/fMRI evidence (Phan et al., 2002; Murphy et al., 2003; Wager et al., 2003), and support that dissociable neural substrates underpin the affective representations of valence. Here, pleasant valence is processed in left IFG (pars Orbitalis), which is partly in agreement with evidence that IFG activity associates to positive stimuli when compared to neutral ones, since IFG activity relates more strongly to negative stimuli when compared to positive ones (Kensinger and Schacter, 2006).

Given that primary but also extrastriate visual activity has functional specialization for emotional images (Lang et al., 1998; Paradiso et al., 1999) and is modulated by valence (Lane et al., 1999; Mourão-Miranda et al., 2003), we report that the activity of right (superior and middle) occipital gyrus and precuneus are modulated by unpleasant stimuli. Unpleasant emotions activate the right extrastriate area and are distinguished from neutral or pleasant ones by the activation of the occipitotemporal cortex (Lane et al., 1999). Our precuneus and secondary visual activations concur with previous evidence (Pourtois et al., 2006; Nielen et al., 2009) and justify that these regions are highly sensitive to unpleasant stimuli.

Caudate nucleus is often activated for romantic love (Bartels and Zeki, 2000) and beauty (Ishizu and Zeki, 2011). Our caudate nucleus activity for unpleasant valence may contradict findings regarding these discrete emotions (positively valenced), but is in line with evidence that the dorsal striatum encodes negative stimuli (George et al., 1995), that the caudate nucleus has larger responses for negative when compared to positive images, and is crucial in withdrawal (Carretié et al., 2009). Since the activity of the regions does not show an exclusive relationship with any of the valence levels, we regard that these can be functionally selective for positivity or negativity. Thus, valence is flexibly encoded across instances by valence-general brain regions in accordance to the valence-general “affective workspace” hypothesis (Barrett and Bliss-Moreau, 2009).

#### Arousal Encoding

The amygdala is an important substrate of emotions, and its constituent nuclei may have diverse functions as discussed in detail in our previous study (Styliadis et al., 2014). The left amygdala is engaged in the detailed evaluation of the stimulus (Markowitsch, 1998), and modulations along arousal (Gläscher and Adolphs, 2003). In our previous study which explored emotional processing in the 2–30 Hz frequency wideband (Styliadis et al., 2014), the laterobasal (LB) sub-division of the right amygdala was activated for unpleasant valence and we speculated that LB activation may unfold via a rapid subcortical route in line with amygdala’s role in processing aversive information (Morris et al., 1998). In the current study, the LB sub-division of the left amygdala was activated at ∼160 ms for high arousal. Our results are in line with the suggestion that the arousal effect between 200 and 300 ms is related to the amygdala’s response to affective stimuli (Olofsson et al., 2008). Overall, there is some evidence that the LB sub-division may be functionally selective for unpleasant and high arousing stimuli at specific temporal windows and this selectivity may also drive its lateralization. Regarding amygdala’s selectivity, the right amygdala may be involved in the initial and rapid, probably automatic, detection of an emotional stimulus, whereas the left amygdala may follow up by performing an elaborate stimulus evaluation but also a specific analysis of variations in the magnitude of arousal associated with the stimulus (Wright et al., 2001; Gläscher and Adolphs, 2003). This pattern for emotional processing (i.e., first valence, then arousal) is evident in the current study and a previous one (Styliadis et al., 2015) which focused on the cerebellar role in emotional processing, and though that in our previous study (Styliadis et al., 2014) on the subdivisions of the amygdala we did not provide the time course of activation, it is plausible that the right LB amygdala was activated for unpleasant valence at very early time intervals, similar to the ones here for unpleasant valence and in line with the roles for left and right amygdala mentioned above (Wright et al., 2001; Gläscher and Adolphs, 2003; Sergerie et al., 2008). Differences in the laterality of amygdala involvement between our previous study (Styliadis et al., 2014) and the current one could also be attributed to the different frequency bands which were analyzed. In addition, the amygdala has strong interconnections with the OFC (here left) (Amaral et al., 1992), and these structures (the basolateral amygdala in specific) have been proposed to form a crucial circuit in processing behaviourally relevant stimuli (Schoenbaum et al., 1998). In contrast to pleasant valence, high arousal was processed in a different region of left IFG, pars Opercularis. Pars Opercularis is correlated with high arousal (Nielen et al., 2009) and is activated when humans attempt to decrease their sexual arousal during erotic films (Beauregard et al., 2001). Our results are similar to those of an fMRI study (Nielen et al., 2009), though no amygdala activity was found there. Also, there are frequent reports that the middle temporal gyrus and the lateral PFC are sensitive to the arousal evoked by emotional images (Mourão-Miranda et al., 2003; Dolcos et al., 2004). Overall, our findings for high arousal support the ventral emotional arousal system that relays information from the amygdala and posterior parietal cortex into ventral PFC (Mega et al., 1997).

### Interaction of Valence and Arousal

Our results for the valence by arousal interactions confirm that some brain regions respond independently to both valence and arousal (Lewis et al., 2007; Styliadis et al., 2014), and suggest that valence and arousal are not fully dissociated in the engaged structures. Our results show that the neuronal responses to high arousing pleasant stimuli are processed in different regions in comparison to responses for high arousing unpleasant stimuli (Nielen et al., 2009). Our IFG (pars Orbitalis and pars Opercularis) findings for the pleasant and high arousal interaction may reflect that emotion regulation processes are a result of the interplay of lateral PFC regions, in line with models which suggest that strategy initiation and application are promoted by these regions (Ochsner et al., 2012). Indeed, the IFG has been linked to the implementation of cognitive control strategies (e.g., reasoning about emotions) (Ochsner and Gross, 2005) as well as in the decision to initiate emotion regulation. The putamen is found to co-activate with the caudate nucleus for romantic love (Bartels and Zeki, 2000) and euphoric situations (Breiter et al., 1997) which can be characterized as pleasant and high arousal. Thus, our dorsal striatum activity may reflect previous evidence for regulation of responses to rewarding stimuli. Recently, the activity of the dorsal striatum was correlated with increased cognitive control, and it was suggested that it indeed mediates cognitive control in decision making (Robertson et al., 2015). It is highly possible that IFG may interact in a context-dependent manner with the left superior frontal gyrus, caudate nucleus and the putamen to carry out cognitive control processes.

### Parallel Spatiotemporal Streams of Emotional Visual Processing Depend on Valence and Arousal

Our data support that valence and arousal neuronal representations unfold in parallel in several regions, along distinct temporal streams, which depend on valence and arousal. In line with event related potentials (ERPs) evidence, valence encoding is prioritized in comparison to the encoding of all other effects (onset at ∼60 ms) and is concluded within 320 ms after the stimulus onset (Olofsson et al., 2008). The observed time sequence for valence ties with evidence showing early and short-lived ERP responses for valence initiating at ∼100 ms (Cuthbert et al., 2000; Codispoti et al., 2006). We demonstrate that pleasant and unpleasant valence not only modulate the activity of valence-general brain regions (Barrett and Bliss-Moreau, 2009) but also evolve along different temporal streams despite the common and very early onset. Pleasant valence is short-lived (offset at ∼110 ms) compared to unpleasant valence (offset at ∼320 ms). The early valence effect is related to the biological significance of not only negative (Baumeister et al., 2001; Olofsson et al., 2008), but also both negative and positive stimuli (Keil et al., 2002; Schupp et al., 2003). The longer duration of processing negative stimuli may be in line with evidence postulating a fundamental influence of valence on memory accuracy. Details of negative information are often vividly remembered than positive information (Ochsner, 2000). This strategy may thus entail increased processing times. Nevertheless, we can only speculate that this is reflected in our findings given the absence of the required behavioral outcomes.

In support to the time sequence of arousal (150–1000 ms duration), ERP responses for arousal usually initiate at ∼300–400 ms, or even at ∼150 ms after the stimulus onset and last for several hundreds of milliseconds (Olofsson et al., 2008). High arousing (both positive and negative) stimuli elicit more pronounced late ERP (e.g., N2, P3) components in comparison to low arousing stimuli, even when the affective stimuli are briefly presented (Keil et al., 2002; Schupp et al., 2003). The arousal effect on late ERP components is thought to represent motivation-driven attention to high arousing stimuli. Therefore, an association of selectivity of visual stimuli in respect to their motivational qualities may require a detailed processing at the late stage of emotional processing (Schupp et al., 2004).

Our results suggest that the valence by arousal interactions take place at both early and subsequent temporal stages of emotional processing (Feng et al., 2014) but only after the process of valence and arousal initiates. There is evidence that arousal modulates valence effects on emotional processing at both behavioral and neural levels (Robinson et al., 2004; Lewis et al., 2007; Nielen et al., 2009). A recent investigation of the temporal profile of valence by arousal interaction employing a similar design and task to ours (Feng et al., 2014) demonstrated that the valence by arousal interaction was observed at 160–190 ms, 220–320 ms, and 400–700 ms. A previous study of the same group employing an implicit and not a passive task found significant interactions between valence and arousal at both early and late stages over both parietal and frontal sites at 100–200 ms, 200–300 ms, and 300–400 ms (Feng et al., 2012). While our results fit well with their findings in terms of the temporal windows observed and the brain regions activated, they provide contrary evidence regarding the pattern of the interactions. These revealed that negative stimuli evoked larger neural responses compared with positive pictures at the high-arousal level and smaller neural responses than positive stimuli matched at low arousal. Our findings are also not consistent with evidence in a study employing a lexical decision task with emotional words along the three levels of valence (unpleasant, neutral, and pleasant) and arousal (low, medium, and high) (Recio et al., 2014). Though there is some agreement that the interactions between valence and arousal take place at early temporal stages of emotional processing (here at late stages as well) they report low and medium arousal modulation for valence effects but not for high arousal as demonstrated here. Notably, in other studies of similar but not identical design and task as Recio et al. (2014), valence and arousal did not interact in ERPs, suggesting independent valence and arousal contributions to emotion effects in word processing (Bayer et al., 2012; Delaney-Busch et al., 2016). It seems that the way arousal and valence interact is more complex than currently acknowledged and there is not much agreement on the patterns of interactions that unfold along the spatiotemporal evolution of emotional processing. Recent studies like Recio et al. (2014) have attempted to explore more regions of the wide spectrum of the valence-arousal continuum, while other studies like our own have focused on distinct regions of this continuum. Consequently, it is plausible that these inconsistencies may originate from the differences between these studies such as their design and task, or the type and number of the stimuli, or more likely the different coverage of the valence-arousal continuum. These gaps in the literature can only be addressed with more detailed experiments (see also Limitations section).

We demonstrate that cortical responses in the frontal, occipital, and parietal cortices for emotional visual processing onset between 60 and 100 ms consistent with previous evidence (Liu and Ioannides, 2010). The evolution of emotional processing unfolds along neural substrates that are located in the ventral (i.e., IFG, occipital gyrus, precuneus, OFC, temporal gyrus), and the dorsal stream (i.e., IFG, superior frontal gyrus, IPL, SPL), but also in subcortical regions. Right IFG serves as an integrator of information received from both the ventral and the dorsal stream (Takahashi et al., 2013). Here, the left IFG was activated for valence and arousal effects, and for their interaction in different temporal windows, likely highlighting IFG’s time-dependent functional selectivity regarding the received input. The quite early response of the orbital region of the IFG (∼60 ms) supports that in the context of emotional-related stimuli, the human brain uses a two-pathway architecture which may operate in parallel and is likely to be functionally selective for different classes of stimuli (Rudrauf et al., 2008). This architecture can account for the rapid extraction of relevant information present in the stimuli before visual processing takes place (Vuilleumier, 2005). Similarly to the conclusions reached by Bar et al. (2006) and Rudrauf et al. (2008), the very early response of the orbital region of IFG may reflect the rapid top-down modulation of visual processing. Future studies employing a functional connectivity analysis could potentially confirm or rule out the possibility that parallel isolated cognitive processes take place independently in substrates located in the dorsal and ventral streams.

### Theoretical Implications

Our findings have implications with theories discussing how multiple levels of affective input influence the processing of emotional pictures or words. In respect to the effect of valence regardless of the level of arousal, many studies converge on a general distinction between valenced (both pleasant and unpleasant) stimuli when compared to neutral stimuli that are matched for low arousal. However, there also reports of larger effects of pleasant than of unpleasant stimuli matched on arousal (Kissler et al., 2009) as well as larger (Ito et al., 1998) or longer (Hajcak and Olvet, 2008) effects of unpleasant than of pleasant high arousal matched stimuli. In addition, there is evidence of an enhanced processing after around 400 ms for high arousing stimuli when compared to low arousing ones, independent of valence (Cuthbert et al., 2000; Schupp et al., 2000). A recent ERP study with a similar design and task to ours reported larger neural responses of negative than of positive stimuli at the high-arousal level and smaller neural responses of negative than of positive stimuli matched at low arousal (Feng et al., 2014). Studies with more comprehensive designs than our own involving a better manipulation of valence (unpleasant, neutral, and pleasant) and arousal (low, and high but also medium, see Recio et al., 2014) have confirmed the processing advantage of pleasant over unpleasant stimuli matched on arousal (Bayer et al., 2012), but have suggested that the arousal effect at around 400 ms is elicited not only by high arousing stimuli when compared to low arousing ones (both pleasant and unpleasant low arousing and neutral) (Bayer et al., 2012; Leite et al., 2012), but also by neutral high arousing in contrast to neutral low and medium arousing stimuli (Recio et al., 2014) (however, this onsets around 275 ms). Relative to our findings around 400 ms (namely high arousal, and interactions between valence and arousal), it is plausible that neutral high arousing stimuli could induce enhanced processing like that for pleasant and unpleasant high arousing stimuli, and thus their comparison would provide a finer characterization of these effects (see also Limitations section).

### Methodological Issues

Magnetoencephalography has a high temporal resolution on the order of milliseconds but a low spatial resolution for source localization. The sensitivity of the spatial resolution is rapidly decreased as a function of the depth of the neural sources (Hillebrand and Barnes, 2002). Hence, MEG’s reliable detectability of deep brain generators that are crucial to multiple brain processes (e.g., emotion) is still a topic in debate. Traditionally, it has been argued that signals from deep subcortical sources would be too weak to be detectable by MEG sensors given that the magnetic field decreases with the square of the distance between the MEG sensor and the neural source (Hämäläinen et al., 1993; Hillebrand and Barnes, 2002). In addition, it has been argued that the complex cytoarchitecture of the deep source (e.g., hippocampus, basal ganglia) may make any detectable activity from this region quasi-null. However, phantom (Papadelis et al., 2007) and human studies (Papadelis et al., 2009) have contributed to this debate by providing evidence that MEG is able to localize deep thalamic activity with an accuracy of 10–15 mm. Importantly, numerous MEG studies have consistently reported activations generated in the amygdala, the hippocampus or the basal ganglia (Attal et al., 2012). In fact, MEG studies on emotions have lent support to the notion that deep sources such as amygdala can be localized accurately despite relatively low signal strength using various source analysis methods (Ioannides et al., 1995; Streit et al., 2003; Rudrauf et al., 2008; Liu and Ioannides, 2010). Importantly, the SAM technique for MEG allows the identification of signals from deep sources. Indeed, studies using SAM have reported robust amygdala signals over time (Luo et al., 2007, 2009, 2010; Cornwell et al., 2008; Styliadis et al., 2014).

### Limitations

A main disadvantage of the current study is that the stimulus characteristics concerning normative ratings for valence and arousal are not perfectly controlled across the four cells of the 2 × 2 design crossing the factors valence and arousal. IAPS is a well-established and widely used database of affect-inducing stimuli. However, when selecting stimulus sets on the basis of the normative ratings and cutting the continuous valence and arousal dimensions into a specific number of categories, the assumption is made that the valence and arousal are orthogonal (i.e., uncorrelated). Here, we found that there are statistically significant differences regarding the valence ratings not only between cells of the factor valence which is an expected result but also between cells of the factor arousal which was not expected. Similarly, there are significant differences regarding the arousal ratings not only between the cells of the factor arousal but also between the cells of the factor valence. In addition, there are significant valence × arousal interactions for valence and arousal ratings. The significant differences reported in **Table 2** violate the assumption that valence and arousal are orthogonal and show that the valence and arousal effects are not well separated. Therefore, some of the interactions reported here in the brain activation data may have resulted from stimuli with a less than optimal control for the two factors of valence and arousal.

Another limitation is that we did not introduce a third level for valence (neutral) and maybe less important for arousal (medium). Neutral valence can characterize stimuli with very low (but not entirely non-existent) motivational significance (everyday objects) (Lebrecht et al., 2012). Certain types of stimuli (e.g., neutral and high arousing) have not traditionally been employed in studies of emotion processing. However, considering the whole range of valence and arousal should potentially allow a more comprehensive investigation of the neural underpinnings of these dimensions independently from each other and their possible interactions. That being said, the majority of ERP studies contrasted emotional (e.g., high arousing positive and/or high arousing negative) with neutral (e.g., low arousing neutral) conditions and showed that the evaluative processing that occurs around 400 ms after stimulus onset and can last for a duration of several hundred milliseconds depends on both context and task (Schupp et al., 2007; Foti and Hajcak, 2008; Dunning and Hajcak, 2009; Hajcak et al., 2009). However, maximizing the contrast between emotional and neutral conditions, produced heterogeneous results as this processing may reflect differences in both valence and arousal, and these results are mixed with respect to the direction of the effects. Taking into account multiple levels of affective input could, for instance, allow the observation of processing advantage for specific combinations of stimuli that would replicate or even extend previous reports of the negativity bias (here evident mainly for high arousal) or positivity offset (Norris et al., 2010) which was less evident here.

As previously noted, most studies, including our own, have explored distinct regions of the valence-arousal continuum providing mixed results. Given that our experimental design allowed us to look only at separate parts (extremes) of the wide range of the valence-arousal spectrum it is plausible that these specific characteristics of the stimuli (i.e., mainly sports and erotica themes in the PHA condition) have influenced the results, especially concerning the very early emergence of positive valence effects and the interactions. Notably, Feng et al. (2014) employed a similar design and task, as well as similar stimuli selection (e.g., PHA condition contains manly sports themes but also erotica) with the current study, and reported a similar evolution of emotional processing; a quite early main effect of valence (90–110 ms), followed by arousal (120–150 ms), and then the interactions. However, the pattern of the interactions as mentioned before (see section “Parallel Spatiotemporal Streams of Emotional Visual Processing Depend on Valence and Arousal” in the Discussion) was different. Whether these inconsistencies in the results are due to methodological and technical issues (design, task, selection, and number of stimuli or even age and gender of participants), or more likely due to limited coverage of distant patches of the valence-arousal continuum (levels of valence and arousal) can only be addressed with more detailed experiments that fill the gaps that now exist in the literature. In close relation, it is plausible that the inclusion of erotic stimuli may have largely influenced the neural responses of the participants as our findings for the interaction of pleasant valence by high arousal may reflect the effort of cognitive control. Our experimental design did not allow to disentangle whether the elicited neural response is the natural emotional response or that of cognitive control entrained by social norm under a non-private context.

In addition, this research is limited by the absence of concurrent measures of emotional responding, on-line experiential ratings, or conventional behavioral outcomes. The stimuli were rated by the participants after each experimental session and their ratings were consistent with the normative ratings of the stimuli. We performed the rating after and not during the MEG recordings to: (i) minimize the contamination of MEG data with muscular artifacts; (ii) limit minor head movements that may affect the accuracy of coregistration; and (iii) avoid interference between the confounding effect of subjective stimulus judgments and the emotion-related responses. Though we did not consider balancing the stimuli in terms of their semantic content, the broad range of our selection offers ecological validity to our protocol, as the same level of valence or arousal can be induced many times by displays of complex events, scenes of natures, or faces and body figures. Nevertheless, this limitation may affect our conclusion regarding the parietal responses to arousing stimuli consistent with the late positive potential. Finally, though the multiple comparison issue in the spatial domain was adequately addressed, the multiple comparison issue across the temporal domain was not as we did not adjust the multiple ANOVA’s *p*-values. Given our small sample size, non-parametric statistics would be more appropriate but would be compromised by the complexity of our experimental design.

## Conclusion

Mapping brain regions for emotional processing tests the probability of activation in brain regions in relation to valence and arousal, but also the probability that the presence of certain levels of valence and arousal induce activations in a given brain region that may be involved in more than one functional circuits. Here, we focused on mapping the emotional related spatial distributions and tracking their time-dependent evolution in milliseconds to demonstrate a complete assessment of emotional processing. We provide an interesting account of the time course of visual emotional processing in the brain using mainly well-established methods. In sum, our findings support the parallel spatiotemporal evolution of emotional processing involving mainly dissociable neural pathways for valence and arousal, even at a very early stage. Despite the relatively small sample size, our findings align well with the notion that the timing of emotional processes is important and must be taken into consideration. We have provided the required methodological transparency for other researchers in the field to reproduce our study in a larger sample and/or to design and perform improved studies to address similar research questions.

## Author Contributions

AI, PB, and CP designed the experimental protocol. CS and CP recorded MEG data. CS performed the data analyses under CP supervision. CS wrote the main manuscript text and prepared **Figures 1–3**. All authors reviewed the manuscript.

## Conflict of Interest Statement

AI works for AAI Scientific Cultural Services Limited (AAISCS) for much of the work that lead to the publication. The remaining authors declare that the research was conducted in the absence of any commercial or financial relationships that could be construed as a potential conflict of interest.

## Footnotes

1 PHA for males: 1720, 1811, 4001, 4006, 4141, 4142, 4150, 4180, 4210, 4220, 4225, 4232, 4240, 4250, 4255, 4290, 4300, 4310, 4311, 4320, 4607, 4608, 4651, 4652, 4658, 4659, 4660, 4664, 4664.1, 4670, 4681, 4683, 8080, 8185, 8186, 8190, 8340, 8400, 8499, 8501. PHA for females: 2150, 2216, 2303, 2345, 2389, 2550, 4460, 4470, 4490, 4503, 4510, 4520, 4532, 4533, 4537, 4538, 4542, 4561, 4572, 4598, 4599, 4607, 4608, 4609, 4611, 4626, 4656, 4659, 4660, 4670, 4680, 4681, 4687, 4689, 4695, 5621, 5629, 7502, 8030, 8034. PLA for males: 1450, 1601, 1610, 1620, 1750, 1812, 1900, 1920, 2050, 2070, 2165, 2170, 2235, 2260, 2299, 2303, 2360, 2370, 2388, 2501, 2530, 2550, 2650, 2660, 4614, 5000, 5001, 5010, 5020, 5030, 5200, 5831, 5891, 7080, 7325, 7340, 7545, 7900, 8330, 8497. PLA for females: 2000, 2010, 2037, 2152, 2260, 2299, 2304, 2311, 2360, 2370, 2388, 2395, 2398, 2501, 2510, 2530, 2540, 2598, 2620, 5000, 5010, 5020, 5030, 5200, 5520, 5551, 5611, 5631, 5711, 5720, 5750, 5760, 5764, 5779, 5800, 5811, 5891, 7039, 7340, 7545. UHA for males: 1525, 2681, 2683, 2688, 2730, 2811, 3000, 3010, 3015, 3030, 3053, 3060, 3068, 3069, 3071, 3080, 3100, 3102, 3110, 3120, 3130, 3150, 6230, 6250.1, 6300, 6313, 6350, 6510, 6540, 6550, 6560, 6570, 8485, 9040, 9252, 9410, 9630, 9635.1, 9810, 9902.

2 http://www.affective-sciences.org/spatfreq

3 http://www.fil.ion.ucl.ac.uk/spm/software/spm8/

4 http://www.fz-juelich.de/inm/inm-1/EN/Forschung/_docs/Gehirnkarten/gehirnkarten_node.html
